# Trigonometric Concept of Fluoroscopy-Guided Percutaneous Renal Access

**DOI:** 10.7759/cureus.8817

**Published:** 2020-06-25

**Authors:** Danny Darlington, Jagatheswaran Chinnathambi, Arunkumar Jamburaj, Kim J Mammen

**Affiliations:** 1 Urology, Pondicherry Institute of Medical Sciences, Pondicherry, IND; 2 Urology, All India Institute of Medical Sciences, Rishikesh, IND

**Keywords:** percutaneous nephrolithotomy, renal access, triangulation method, trigonometry

## Abstract

Percutaneous nephrolithotomy is the standard surgical management of large renal calculi. Percutaneous renal access using the triangulation method has been an enigma for the endourologist to master and teach. This surgical conundrum is due to the uncertainty in the angle and depth required to puncture the target calyx. We describe a novel trigonometric method of renal access where both the angle and the depth of puncture are easily determined before the puncture.

## Introduction

Percutaneous nephrolithotomy (PCNL) is the accepted treatment of choice for large renal calculi and staghorn stones [1]. An optimal percutaneous renal access is paramount in achieving good surgical outcomes in PCNL. Several methods of fluoroscopy and ultrasound-guided renal access have been described in the literature [2]. Fluoroscopy-guided renal access can be achieved using either the triangulation or bull’s eye method [3]. While the triangulation method is versatile, it is difficult to master as well as teach to a novice surgeon. On the other hand, the bull’s eye method is relatively straightforward but associated with an increased risk of radiation exposure to the hands of the surgeon. However, each technique has its advantages and disadvantages [4]. We describe a novel trigonometric method to determine the angle and depth of renal puncture thereby obtaining straightforward renal access during prone PCNL with a significantly short fluoroscopy time.

## Technical report

The salient feature of the trigonometric concept of the PCNL puncture technique is that the angle and depth of puncture are predetermined and fixed, thereby facilitating rapid renal puncture. This concept is ideally applied for the triangulation method of percutaneous renal access in prone PCNL.

**Figure 1:**
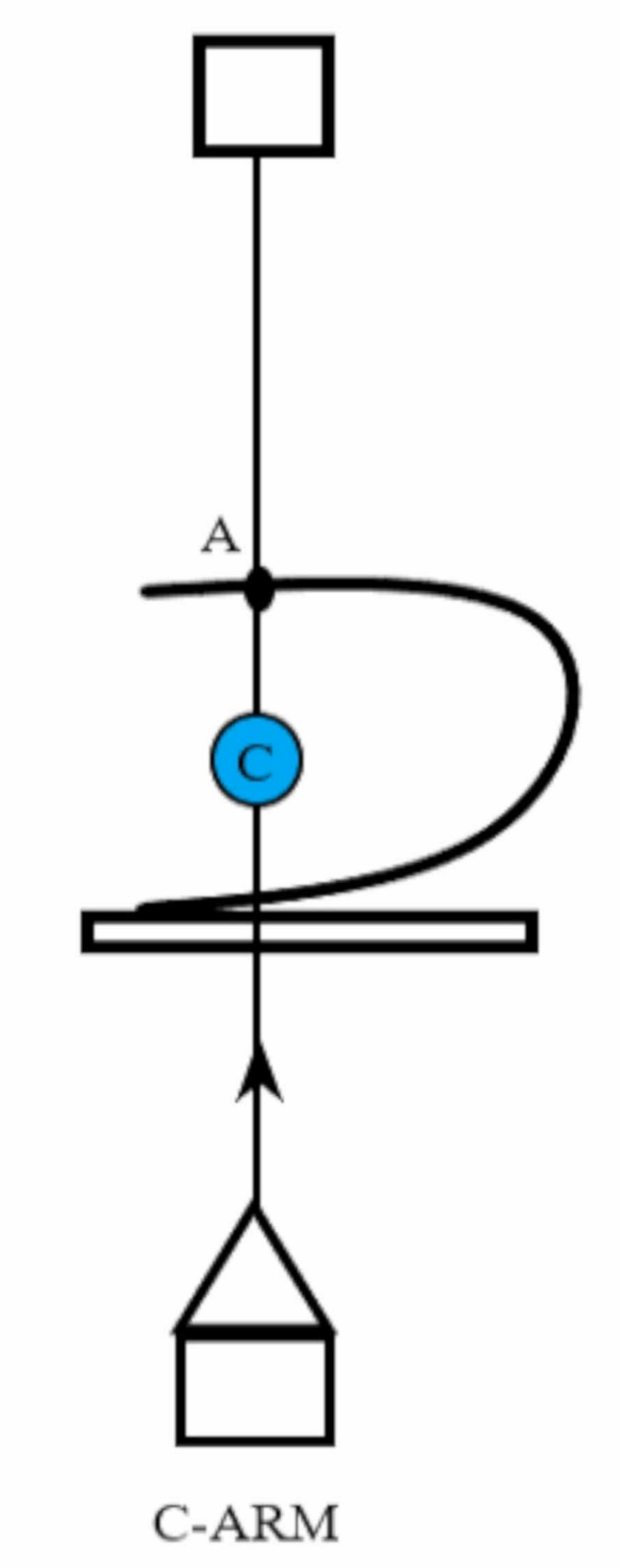
With the fluoroscopy at zero degrees, point A is marked on the skin corresponding to the target calyx C

**Figure 2:**
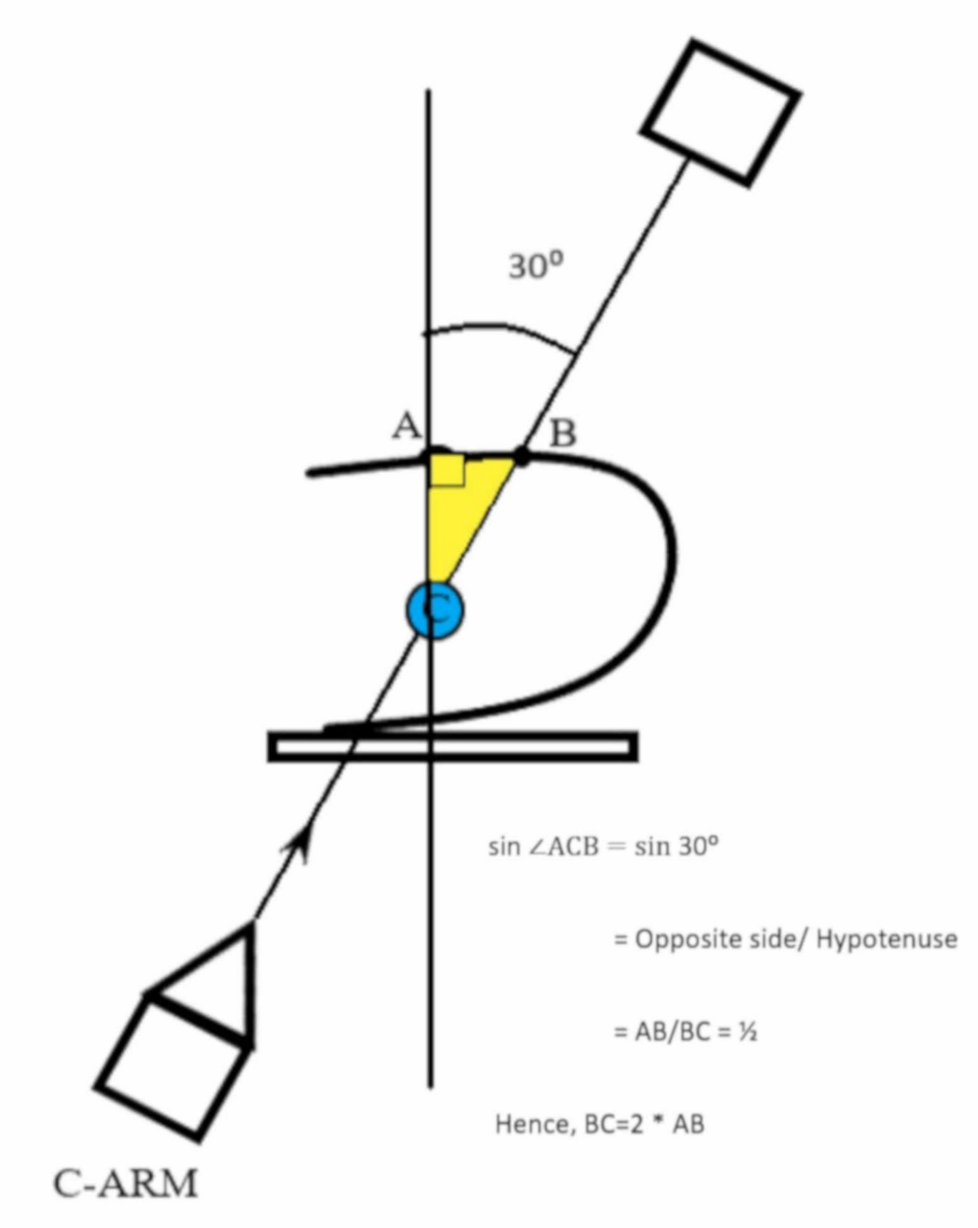
With the fluoroscopy at thirty degrees, point B is marked on the skin overlying the target calyx C forming a right-angled triangle

With the patient in the prone position and the fluoroscopy at zero degrees, point A is marked on the skin corresponding to the targeted calyx C (Figure 1). With the fluoroscopy rotated 30 degrees towards the surgeon, point B is then marked on the skin (Figure 2). The line connecting points A and B on the skin almost forms a right angle to the line connecting points A and C, especially in obese patients with a flat loin. Although the angle may not be exactly 90 degrees in lean patients due to the convexity of the loin, the difference is negligible. The angle ACB is equal to 30 degrees, which is equal to the degree of rotation of the fluoroscopy (Figure 2).

**Figure 3:**
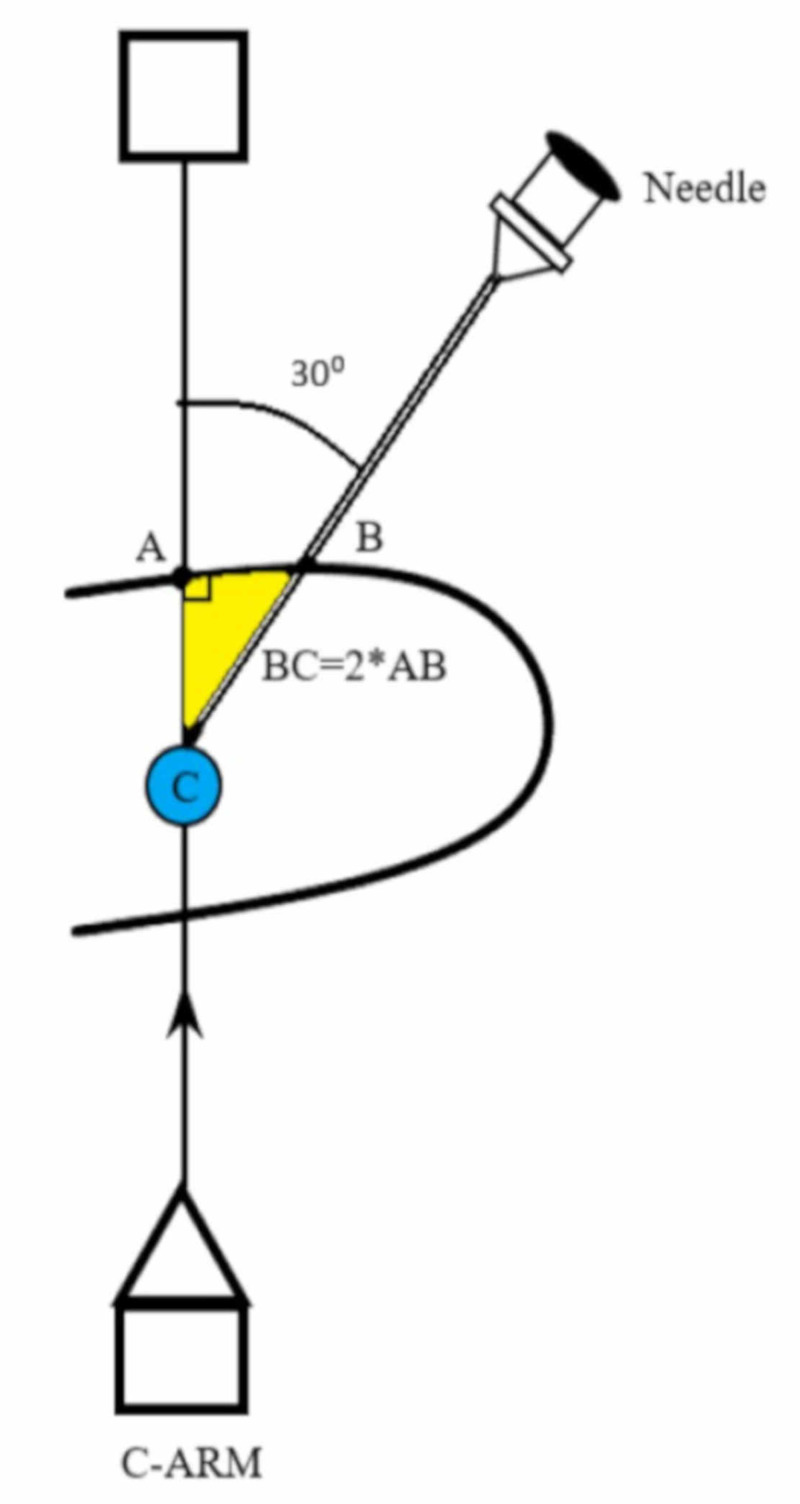
The fluoroscopy is brought back to zero degrees and the puncture needle is advanced at thirty degrees to a depth equal to twice distance AB to reach the target calyx C

The points A, B, and C form a right-angled triangle at A with the angle at C being thirty degrees and AB the side opposite to this angle at point C. The depth of puncture is BC, which forms the hypotenuse of this triangle. This depth can be calculated from AB by employing the sine trigonometric function (Figure 2). Based on this calculation the depth of puncture is twice the distance AB and the direction is along BC with the angle of puncture 30 degrees from the sagittal plane (Figure 3).

In practice, after marking points A and B on the skin as described above, the distance AB is measured and a marking is made on the graded puncture needle at a distance twice that of AB. This is equal to BC and corresponds to the depth of puncture. The fluoroscopy is then moved back to zero degrees and the puncture needle is advanced at point B, 30 degrees from the sagittal plane. The needle tip enters the targeted calyx (C) when the marker in the puncture needle reaches the skin surface. The use of fluoroscopy at zero degrees serves as an additional monitor to confirm the needle trajectory. The guidewire is placed and the standard steps of PCNL are completed.

This novel trigonometric concept can also be extended to percutaneous renal punctures at 45 degrees. However, it requires the fluoroscopy to be brought to the extreme rotation of 45 degrees and the multiplication factor is 1.414 to calculate the depth of puncture. Another disadvantage is that the axis of puncture at 45 degrees may be offset from the axis of calyx and infundibulum. Hence, the renal access with the needle at 30 degrees, which is along the axis of the target calyx is preferred in most of the cases. Employing this trigonometric concept of the triangulation method, the author has obtained optimal percutaneous access at a single attempt puncture for 40 cases of prone PCNL with a 92.5% success rate, with three patients requiring a second attempt. The mean fluoroscopy time for marking the points A and B is 25 to 30 seconds whereas the mean fluoroscopy time for puncturing the targeted calyx is 60 to 80 seconds. These short fluoroscopy times were achieved due to the fixed angle and predetermined depth of puncture. There was no incidence of access-related vascular or organ injury.

## Discussion

Percutaneous nephrolithotomy (PCNL) is widely regarded as the standard surgical management option of large renal calculi. Although several authors have introduced modifications of tract size to ameliorate the morbidity associated with the procedure, obtaining ideal percutaneous access is exceedingly important in achieving good outcomes [5]. Percutaneous renal access is usually achieved using either ultrasound or fluoroscopy guidance [6-7]. Fluoroscopic-guided punctures can be made employing triangulation, the bull’s eye method, or a combination of both [3]. Although both techniques are equivalent in terms of patient outcomes, they pose unique challenges to the novice surgeons and the bull’s eye technique is easier learned than the triangulation method [5].

While the triangulation method provides the best possible puncture, it is difficult to master as acquiring the depth assessment skill remains the rate-limiting step in learning this technique [8]. Another advantage of this method is the lower radiation exposure to the hands of the surgeon compared to the bull’s eye technique. The bull’s eye method is straightforward and is readily mastered by novice surgeons. However, it is associated with more radiation exposure to the hands of the surgeon as per the European Section of Urotechnology [9]. This method is also associated with torque on the renal parenchyma predisposing to bleeding.

Li et al. have designed a stereotactic localization system consisting of a special custom-made ruler and a goniometer to measure the depth and angle of puncture. Their method is associated with lesser fluoroscopy times than the conventional method. However, it requires a special patented ruler and goniometer, which is cumbersome [10]. Gyanendra et al. describe a method of determining the angle and depth of puncture using the universal triangle solver software. However, this requires the bull’s eye technique to assess the angle of calyx and a smartphone to perform the calculations [11].

The main hurdle in learning the triangulation method is the difficulty in assessing the depth of puncture. This happens because both the angle and depth are to be monitored in different planes requiring constant adjustments of the fluoroscopy. The trigonometric concept avoids this by determining and fixing both these variables required for a successful puncture. Hence, it is not only easier to master but also to teach novice surgeons. This method will theoretically reduce the fluoroscopy times and radiation exposure to hands although further studies are required to ascertain these advantages. Another salient feature of this technique is that the angle and depth of puncture and thereby the needle trajectory are easily determined on the table without the need for sophisticated instrumentation or complex calculations. The mean fluoroscopy time is short in our cohort of patients using this technique, which is rarely achieved in the early part of learning the conventional triangulation method. The theoretical disadvantage of this technique is the fixed angle of puncture, which may not be ideal in malrotated kidneys although in such cases, the puncture can be tried at 45 degrees. This method may not be suitable for supine PCNL given the extreme angulation of fluoroscopy and puncture needle in the supine position, which is further hindered by the operating table.

## Conclusions

Percutaneous nephrolithotomy is the standard of care for renal stones and hence it is vital for young urologists to master this procedure in the present era of endourological management of urolithiasis. The choice between the triangulation and bull’s eye technique for percutaneous renal access is mainly the preference of the surgeon as each method has its own advantages and disadvantages. The trigonometric concept of the triangulation method is a novel technique that requires no specialized additional instrumentation or complex calculations in achieving percutaneous renal access with short fluoroscopy time. This straightforward method also facilitates surgeons to master and teach the triangulation technique of renal access with ease.

## References

[1] Zumstein V, Betschart P, Abt D, Schmid HP, Panje CM, Putora PM (2018). Surgical management of urolithiasis - a systematic analysis of available guidelines. BMC Urol.

[2] Ng FC, Yam WL, Lim TYB, Teo JK, Ng KK, Lim SK (2017 Sep). Ultrasound-guided percutaneous nephrolithotomy: advantages and limitations. Investig Clin Urol.

[3] Sharma GR, Luitel B (2019). Techniques for fluoroscopy-guided percutaneous renal access: an analytical review. Indian J Urol.

[4] Budak S, Yucel C, Kisa E, Kozacioglu Z (2018). Comparison of two different renal access techniques in one stage percutaneous nephrolithotomy: triangulation versus eye of the needle. Ann Saudi Med.

[5] Tepeler A, Armağan A, Akman T (2012). Impact of percutaneous renal access technique on outcomes of percutaneous nephrolithotomy. J Endourol.

[6] Chu C, Masic S, Usawachintachit M (2016). Ultrasound-guided renal access for percutaneous nephrolithotomy: a description of three novel ultrasound-guided needle techniques. J Endourol.

[7] Chan CJ, Srougi V, Tanno FY, Jordão RD, Srougi M (2015). Percutaneous puncture of renal calyxes guided by a novel device coupled with ultrasound. Int Braz J Urol.

[8] Sharma GR, Maheshwari PN, Sharma AG, Maheshwari RP, Heda RS, Maheshwari SP (2015). Fluoroscopy guided percutaneous renal access in prone position. World J Clin Cases.

[9] Kyriazis I, Liatsikos E, Sopilidis O, Kallidonis P, Skolarikos A (2017). European section of urotechnology educational video on fluoroscopic guided puncture in percutaneous nephrolithotomy: all techniques step by step. BJU Int.

[10] Li X, Liao S, Yu Y, Dai Q, Song B, Li L (2012). Stereotactic localisation system: a modified puncture technique for percutaneous nephrolithotomy. Urol Res.

[11] Sharma G, Sharma A (2015). Determining the angle and depth of puncture for fluoroscopy-guided percutaneous renal access in the prone position. Indian J Urol.

